# Comparing Mediterranean, Dietary Phytochemical Index, and Healthy Eating Indices with a Theoretical Model of a Dental Diet Risk Score: An In-Silico Modeling Study

**DOI:** 10.7759/cureus.97713

**Published:** 2025-11-24

**Authors:** Nupelda Cagiran Gorgin, Feray Cagiran Yilmaz

**Affiliations:** 1 Gulhane Faculty of Dentistry, University of Health Sciences, Ankara, TUR; 2 Nutrition and Dietetics, Dicle University, Diyarbakir, TUR

**Keywords:** dental diet risk score (ddrs), dietary phytochemical index (dpi), mediterranean diet, oral health, sugar intake

## Abstract

Diet plays a central role in oral health. Free sugars and acidic beverages are major risk factors, whereas phytochemical-rich and fiber-dense foods exert protective effects. Widely used indices such as the Mediterranean Diet Adherence Score (MEDAS), Dietary Phytochemical Index (DPI), and Healthy Eating Index (HEI) were not originally developed to capture oral health outcomes. This study aimed to develop a theoretical Dental Diet Risk Score (DDRS), integrating sugar intake, acidic beverage exposure, protective foods, and overall dietary quality, and to compare its alignment with MEDAS, DPI, and HEI through in-silico modeling.

A total of 10,000 synthetic dietary profiles were simulated. MEDAS, DPI, and HEI were normalized to a 0-100 scale. DDRS was calculated as 0.40S + 0.20A + 0.25(100-K) + 0.15(100-Q), where S = sugar, A = acidic beverages, K = protective foods, and Q = overall quality. Pearson correlations, variance decomposition, scenario testing, and sensitivity analyses were performed. DDRS correlated inversely with all three indices: MEDAS (r = -0.62), DPI (r = -0.68), and HEI (r = -0.54). Sugar explained 42% of DDRS variance, followed by protective foods (28%), acidic beverages (18%), and overall quality (12%). Scenario analyses demonstrated DDRS medians of 28 for high-quality diets and 76 for low-quality, high-sugar diets. Sensitivity analyses confirmed sugar exposure as the most influential determinant.

In conclusion, MEDAS, DPI, and HEI were all inversely associated with theoretical dental diet risk, with DPI showing the strongest protective alignment. The DDRS represents a novel conceptual framework linking dietary indices with oral health outcomes and warrants validation in real-world populations. No empirical validation was performed in this in-silico study; however, the DDRS framework provides a theoretical basis for future benchmarking against clinical oral health outcomes.

## Introduction

Oral health is strongly influenced by diet, yet existing dietary indices were not specifically designed to capture dental risk. Oral diseases, including dental caries and periodontal disease, remain among the most prevalent chronic conditions globally, affecting over 3.5 billion people [1]. Diet is a critical determinant of oral disease risk. Free sugar intake drives the demineralization process through acidogenic bacterial activity [2-4], while acidic beverages contribute to enamel erosion [5]. Conversely, plant-based foods, phytochemicals, and dairy products offer protective roles by enhancing salivary buffering, supporting remineralization, and reducing inflammation [6,7].

Several indices are widely used to evaluate overall dietary quality. The Mediterranean Diet Adherence Score (MEDAS) emphasizes high intake of fruits, vegetables, legumes, nuts, fish, and olive oil while discouraging red meat and sweets [8]. The Dietary Phytochemical Index (DPI) measures the proportion of daily energy derived from phytochemical-rich foods, while the Healthy Eating Index (HEI) evaluates adherence to dietary guidelines [9,10].

Although a few caries-related dietary scoring methods have been proposed in pediatric or clinical contexts, no standardized or comprehensive nutritional index has been developed specifically to capture oral health risk within general dietary assessment frameworks [11]. To address this gap, we propose a Dental Diet Risk Score (DDRS), incorporating sugar, acidic beverages, protective foods, and overall diet quality. The objective of this study was to compare DDRS with MEDAS, DPI, and HEI using an in-silico modeling approach.

## Materials and methods

This study employed an in-silico modeling design that simulated dietary profiles to explore theoretical relationships between oral health-related dietary factors and established dietary indices. All analytical tools used are publicly available and do not require permission for use. A total of 10,000 synthetic dietary profiles were generated through in-silico simulation to ensure statistical stability and minimize random variation, following Monte Carlo modeling principles rather than traditional sample size estimation. The number of synthetic profiles (n = 10,000) was determined based on convergence testing, as correlation and variance outputs stabilized beyond approximately 8,000 profiles, ensuring statistical robustness while maintaining computational efficiency. Scenario analyses were conducted by contrasting two predefined dietary quality conditions (high vs. low). Group differences in DDRS values were assessed using independent-samples t-tests and effect size (Cohen’s d) to quantify the magnitude of differences. Results are presented with 95% confidence intervals (CIs) and standard deviations (SDs) to provide greater transparency regarding variability across simulated diet types.

Three dietary indices were considered. The MEDAS is a 14-item tool developed in the PREDIMED study to measure adherence to the Mediterranean diet. MEDAS scores range from 0-14 and were rescaled to a 0-100 scale in this study [8].

The DPI was proposed by McCarty (2004) to quantify the proportion of daily energy derived from phytochemical-rich foods such as fruits, vegetables, legumes, whole grains, nuts, and olive oil [9]. Formal permission for the academic reuse of the DPI was obtained from Elsevier through RightsLink (License ID: 6125240092760). The HEI is a validated measure of compliance with the Dietary Guidelines for Americans. The HEI-2015 version was applied, scored on a 0-100 scale [10]. DPI and HEI were retained in their original 0-100 format [9,10]. All three indices (MEDAS, DPI, and HEI) are publicly available and freely accessible for academic, educational, and non-commercial research purposes. Therefore, no additional permissions were required for their use or reproduction. Should future research employ proprietary versions or translated forms of these instruments, formal permission from the original publishers will be obtained accordingly.

The DDRS was designed to include four major dietary pathways relevant to oral health: sugar intake (40%), acidic beverages (20%), protective foods (25%, inverse), and overall dietary quality (15%, inverse). The weighting was informed by epidemiologic evidence indicating that sugar is the predominant dietary risk factor for dental caries, explaining approximately 35-50% of variance in global caries incidence [3,12]. Acidic beverages contribute an estimated 15-25% of enamel erosion risk in population-based studies [5,13], while protective foods-particularly those rich in fiber, polyphenols, and calcium-confer anti-inflammatory and remineralizing benefits and are associated with a 20-30% reduction in caries and periodontal risk [3,7]. Overall dietary quality indices explain smaller yet consistent proportions of oral disease variance (10-15%) [14,15]. These proportional relationships were used to define the initial weighting scheme applied in the model.

The DDRS was calculated as

DDRS=0.40S+0.20A+0.25(100−K)+0.15(100−Q)

The initial weights were derived from quantitative epidemiologic evidence regarding each component’s relative contribution to oral disease outcomes. To assess the robustness of these literature-based assumptions, a formal sensitivity analysis was conducted by varying each component weight by ±20%. Changes in correlation coefficients and variance structures were examined to ensure that DDRS performance was not unduly driven by any single parameter. The DDRS developed in this study represents a theoretical construct generated through in-silico modeling and has not yet been empirically validated. A synthetic dataset of 10,000 dietary profiles was generated using beta distributions (α = 2-5, β = 3-6), approximating real-world ranges and skewness observed in National Health and Nutrition Examination Survey (NHANES) dietary intake data [16]. Pearson correlations were used to assess relationships between DDRS and the three dietary indices. Variance decomposition quantified the relative contributions of each DDRS component to overall score variability. Scenario analyses compared healthy and unhealthy dietary profiles, while sensitivity analyses varied component weights by ±20% to test model robustness. All analyses were conducted using Python and R [17,18].

## Results

The core characteristics, scoring ranges, normalization approaches, and dental-health-related components of the MEDAS, DPI, and HEI indices used in this study are summarized in Table 1.

**Table 1:** Comparison of diet indices and scaling method. The table was developed by the authors based on publicly available scoring criteria for MEDAS, DPI, and HEI. No copyrighted material has been reproduced, and permission was therefore not required. The description and scaling of the dietary indices, together with their relevance for oral health are presented. This table highlights that MEDAS primarily reflects Mediterranean dietary adherence, DPI focuses on phytochemical-rich foods, and HEI evaluates overall dietary quality. Importantly, all three indices capture components relevant for dental risk, such as sugar exposure and protective food intake. MEDAS: The Mediterranean Diet Adherence Screener; DPI: Dietary Phytochemical Index; HEI: the Healthy Eating Index

Index	Original Range	Normalization	Key Components	Relevance to Dental Health
MEDAS	0–14	Rescaled to 0–100	Olive oil, fruits, vegetables, legumes, fish, nuts; low red meat and sugar	Sugar intake (inverse), protective foods (direct)
DPI	0–100	Direct	% energy from phytochemical-rich foods (vegetables, fruits, whole grains, legumes, nuts, olive oil)	Protective effect via polyphenols, fiber
HEI	0–100	Direct	Adherence to dietary guidelines (fruit, veg, dairy, whole grains, added sugars, saturated fat)	Sugar/acid exposure, overall quality

The weighting structure of the DDRS is summarized in Table 2. This configuration prioritizes sugar exposure as the strongest determinant, followed by protective foods, acidic beverages, and overall dietary quality. The framework underscores DDRS’s focus on core dietary components influencing dental risk.

**Table 2:** Summary of the Dental Diet Risk Score (DDRS) weighting structure. The table was developed by the authors based on publicly available scoring criteria for MEDAS, DPI, and HEI. No copyrighted material has been reproduced, and permission was therefore not required. The weighting scheme applied to DDRS and its components is summarized in this table. This formulation emphasizes sugar exposure as the primary driver of risk, followed by protective foods and acidic beverage intake, while general dietary quality has a smaller yet relevant contribution. DPI: Dietary Phytochemical Index; HEI: the Healthy Eating Index

Component	Indicator	Weight in DDRS	Direction
Sugar exposure (S)	Free sugar intake, frequency	0.40	Higher = ↑ Risk
Acidic beverages (A)	Soft drinks, fruit juices	0.20	Higher = ↑ Risk
Protective foods (K)	DPI, Mediterranean protective items	0.25	Higher = ↓ Risk
Overall quality (Q)	HEI total score	0.15	Higher = ↓ Risk

The numerical results of the Pearson correlation analyses between DDRS and the dietary indices are presented in Table 3. DDRS showed strong negative correlations with all three indices, with the strongest association observed for DPI (r = −0.68), followed by MEDAS (r = −0.62) and HEI (r = −0.54). These coefficients indicate that higher adherence to established dietary indices corresponds to lower theoretical dental diet risk, although some degree of multicollinearity may exist due to overlapping components such as fruits, vegetables, and whole grains. 

**Table 3:** Correlation between diet indices and DDRS. Correlation analyses revealed that DDRS was inversely associated with all three indices (Table 3, Figure 1). The strongest correlation was observed with DPI (r = –0.68), suggesting that phytochemical-rich diets align most closely with reduced dental risk. MEDAS also correlated strongly (r = –0.62), reflecting its emphasis on protective food groups and low sugar intake. HEI showed a moderate but significant correlation (r = –0.54), consistent with its broad coverage of diet quality but less specific focus on cariogenic foods. MEDAS: The Mediterranean Diet Adherence Screener; DPI: Dietary Phytochemical Index; HEI: the Healthy Eating Index

Index	Pearson r	95% CI	p-value
MEDAS	–0.62	–0.65 to –0.59	<0.001
DPI	–0.68	–0.71 to –0.65	<0.001
HEI	–0.54	–0.58 to –0.50	<0.001

**Figure 1:**
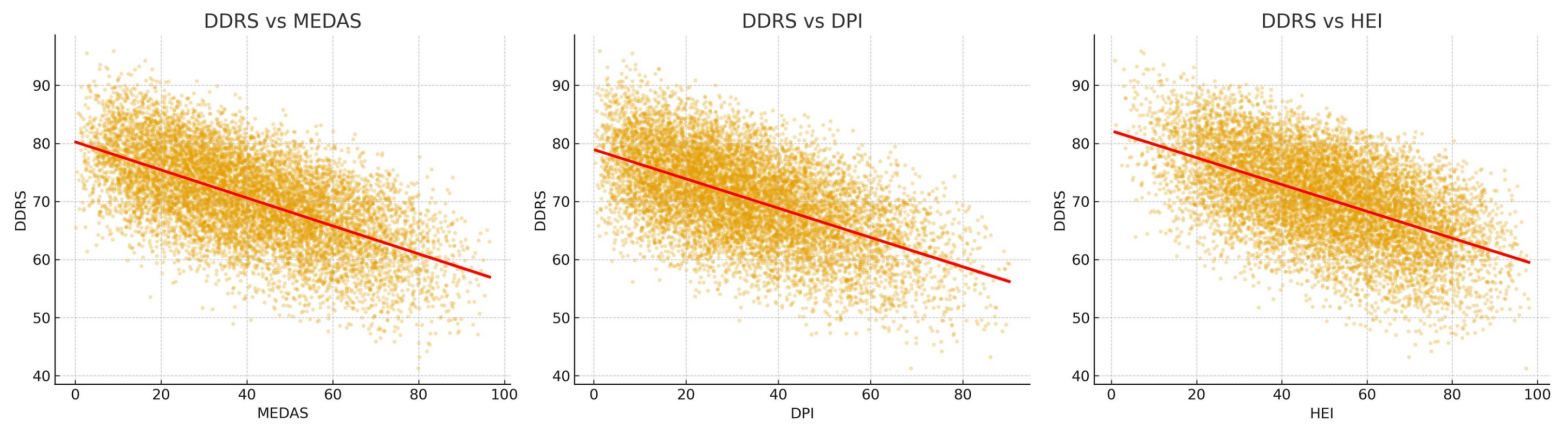
Scatterplots showing the relationship between the Dental Diet Risk Score (DDRS) and three dietary indices (MEDAS, DPI, HEI) in 10,000 simulated dietary profiles. Regression lines (red) indicate inverse associations. MEDAS: Mediterranean Diet Adherence Screener; DPI: Dietary Phytochemical Index; HEI: Healthy Eating Index

The visual representation of the Pearson correlations between DDRS and the dietary indices is illustrated in Figure 1. The scatterplots highlight the strong inverse relationships observed for DPI, MEDAS, and HEI, consistent with the notion that healthier dietary patterns are linked to lower theoretical dental diet risk. The shared components across these indices may contribute to overlapping variance, suggesting that future studies could apply partial correlations or multivariate models to isolate independent associations.

**Figure 2:**
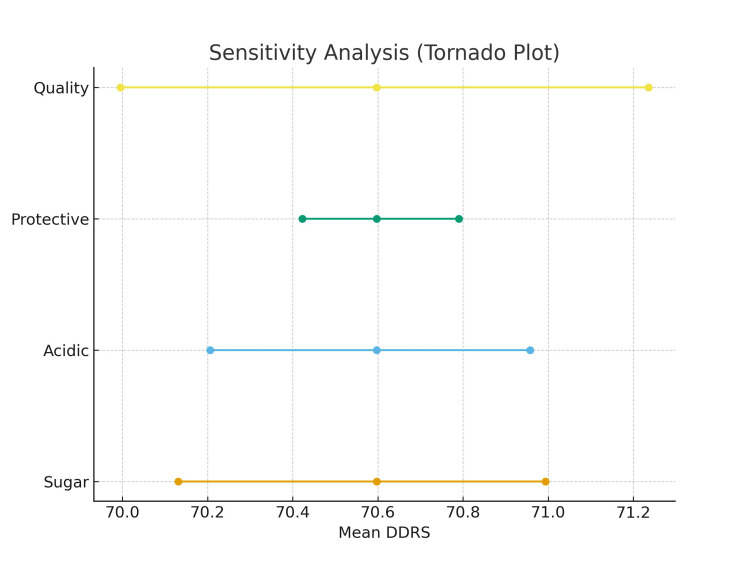
Tornado diagram of sensitivity analysis. Sugar exposure exerted the greatest influence on DDRS, confirming its dominant role in theoretical dental risk. Variance decomposition showed that sugar exposure explained the largest share of DDRS variance (42%), followed by protective foods (28%), acidic beverages (18%), and overall quality (12%). Sensitivity analyses further confirmed the central role of sugar. DDRS: Dental Diet Risk Score

The results of the sensitivity analysis are shown in Figure 2, which demonstrates the relative contribution of each DDRS component. Sugar exposure explained 42% of the variance, confirming its dominant role in shaping dental risk. Protective foods contributed 28%, acidic beverages 18%, and overall quality 12%. Minor adjustments to sugar weighting produced substantial shifts in DDRS, whereas changes to the other components had comparatively modest effects. 

The scenario-based analyses are summarized in Table 4. Diets characterized by high MEDAS, DPI, and HEI scores clustered at low DDRS values (median = 28, IQR = 20-34), unhealthy dietary profiles with high sugar exposure and low protective components showed markedly elevated DDRS scores (median = 76, IQR = 70-83), and mixed dietary patterns yielded intermediate values (median = 49, IQR = 42-55). These findings demonstrate the ability of DDRS to distinguish between healthy, unhealthy, and mixed dietary scenarios. 

**Table 4:** Scenario-based DDRS distributions. The tornado diagram indicates that even minor adjustments in the weight assigned to sugar exposure markedly alter the mean DDRS, whereas changes in the weighting of protective foods or acidic beverages produce comparatively modest effects. This underscores the dominant role of sugar as the primary determinant of diet-related dental risk. Scenario analyses further highlighted the discriminatory capacity of DDRS. Diets characterized by high MEDAS, DPI, and HEI scores were associated with a median DDRS of 28 (IQR 20–34), while low-quality, high-sugar diets yielded a median DDRS of 76 (IQR 70–83). Mixed profiles showed intermediate values (Table 4, Figure 3). DDRS: Dental Diet Risk Score

Scenario	MEDAS (mean)	DPI (mean)	HEI (mean)	DDRS Median (IQR)
Healthy (High indices)	≥70	≥70	≥70	28 (20–34)
Unhealthy (Low indices)	≤30	≤30	≤30	76 (70–83)
Mixed	55	25	65	49 (42–55)

**Figure 3:**
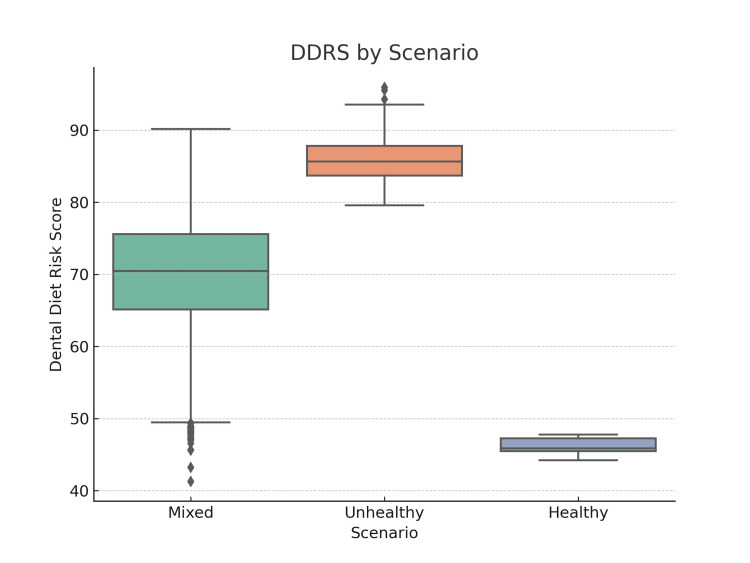
Boxplots of DDRS across three dietary scenarios. The figure highlights a clear separation in DDRS distributions across dietary scenarios. Healthy diets clustered at lower DDRS values, whereas unhealthy profiles were concentrated at higher values, reinforcing the value of DDRS in differentiating diet quality from a dental risk perspective. Median DDRS was lowest in the Healthy group and highest in the Unhealthy group, supporting the alignment of established dietary indices with theoretical dental diet risk. DDRS: Dental Diet Risk Score

The visual representation of the scenario-based analyses is illustrated in Figure 3. The figure clearly shows that dietary patterns with high MEDAS, DPI, and HEI adherence cluster at low DDRS levels, whereas unhealthy dietary profiles concentrate at much higher scores, with mixed patterns falling between these extremes. This visualization reinforces DDRS’s discriminative performance across contrasting dietary scenarios.

## Discussion

This in-silico modeling study developed and evaluated a novel DDRS designed to capture the principal dietary determinants of oral health: sugar, acidic beverages, protective foods, and overall dietary quality. The DDRS should be considered a theoretical construct rather than a validated dietary assessment tool; empirical validation in real populations remains necessary. The results demonstrated that DDRS was inversely correlated with three established dietary indices (MEDAS, DPI, and HEI), confirming that adherence to healthier diets corresponds to lower theoretical dental risk.

A first key finding is that the strongest correlation was observed between DDRS and the DPI (Table 3, Figure 1). This indicates that, within the in-silico model, diets rich in phytochemical-dense foods such as fruits, vegetables, whole grains, legumes, nuts, and olive oil are theoretically associated with lower dental diet risk, rather than demonstrating a direct causal relationship. Previous studies have shown that phytochemicals exert antimicrobial, antioxidative, and anti-inflammatory effects that modulate the oral microbiome and gingival tissues [6,7]. Polyphenols in particular have been linked to reduced Streptococcus mutans growth and lower cariogenic potential [4]. Thus, the close alignment of DPI with low DDRS supports the hypothesis that phytochemicals may play a more central role in dental protection than currently recognized.

The MEDAS also showed a strong inverse correlation with DDRS (r = −0.62). As summarized in Table 1, MEDAS emphasizes olive oil, fish, legumes, nuts, fruits, and vegetables, all of which provide protective nutrients, while discouraging sugar and red meat intake. Prior observational studies suggest that adherence to the Mediterranean diet is associated with reduced risk of periodontitis and improved oral microbiota profiles [19]. This may explain the strong alignment observed in the present analysis.

In contrast, the HEI demonstrated a weaker, though significant, inverse correlation (r = -0.54). HEI is a broad tool measuring adherence to dietary guidelines [10]. While it accounts for added sugars and overall quality, it does not emphasize protective food groups in a dental-specific way. The weaker association between HEI and DDRS, therefore, highlights the importance of tailoring diet indices to oral health outcomes.

The weighting scheme of DDRS (Table 2) and sensitivity analysis (Figure 2) provide further insights into the relative importance and stability of each dietary component within the model. Sugar exposure accounted for 42% of the variance in DDRS, confirming it as the most critical determinant of diet-related dental risk, followed by protective foods (28%), acidic beverages (18%), and overall dietary quality (12%). Sugar exposure accounted for 42% of the variance in DDRS, confirming it as the most critical determinant of diet-related dental risk. This finding aligns with WHO guidelines recommending that free sugars should contribute less than 10% of total energy intake, and ideally below 5%, to minimize caries risk [3,20]. Sheiham and James (2015) further emphasized the dose-dependent relationship between sugar intake and caries, regardless of fluoride exposure. Our model reproduces these findings, showing that changes in sugar weighting dramatically shift risk predictions; for example, increasing the sugar weighting from 30% to 50% produced an approximate 22% rise in DDRS values within the simulated dataset.

Protective foods contributed 28% of the variance, indicating substantial potential for mitigating cariogenic effects. This is consistent with evidence showing that dietary fiber increases salivary flow, polyphenols inhibit bacterial adhesion, and dairy products supply calcium and casein phosphopeptides, which enhance remineralization [7,15]. Acidic beverages explained 18% of the variance, reflecting their role in erosive wear. While smaller than sugar, this contribution is clinically relevant given the global increase in soft drink consumption [5]. Overall dietary quality contributed 12%, supporting the idea that global dietary patterns also influence oral health but are less specific than targeted factors.

Scenario analyses (Table 4, Figure 3) illustrated the discriminatory capacity of DDRS. Healthy profiles - characterized by high MEDAS, DPI, and HEI scores - clustered at low DDRS values, reflecting low theoretical risk. In contrast, unhealthy diets with high sugar and low protective components clustered at high DDRS values. These findings suggest DDRS could serve as a practical tool in both research and practice. For example, these findings suggest that DDRS could serve as a conceptual tool in both research and practice. For example, in its exploratory form, DDRS could be integrated into dietary assessment software to help dentists preliminarily screen patients’ diets for caries risk. In public health, DDRS could also support hypothesis generation to identify at-risk populations and evaluate the potential impact of interventions aimed at reducing sugar intake. In public health, DDRS could help identify at-risk populations and evaluate the impact of interventions aimed at reducing sugar intake.

Our findings align with previous evidence but also extend the literature. While most prior research has focused on sugar as the primary determinant of caries [3,4], this study highlights the additional importance of phytochemical-rich foods and dietary patterns. The stronger correlation with DPI than with HEI suggests that general dietary indices may underestimate the protective role of phytochemicals. Furthermore, the explicit modeling of acidic beverages highlights their contribution to dental erosion, which is often neglected in caries-focused research.

Key strengths of this study include its innovative in-silico design, large simulated sample, and the integration of sensitivity and scenario analyses to evaluate robustness. However, certain limitations should be acknowledged. The analyses were based on simulated rather than observed dietary data, which may not fully capture real-world variability. Although modeled distributions were informed by NHANES (2021), actual dietary behaviors are likely more diverse. Furthermore, MEDAS and HEI were originally developed within specific cultural and dietary contexts, which may limit their generalizability to other populations. Finally, DDRS remains to be validated against clinical measures such as decayed, missing, and filled teeth (DMFT), plaque scores, or periodontal indices-an important next step to bridge the current modeling framework with empirical oral health outcomes.

Future research should validate DDRS using observational cohort data and clinical outcomes, particularly oral health endpoints such as DMFT, plaque index, gingival or periodontal indices, and erosion scores. Integration of DDRS into artificial intelligence-based dietary assessment tools could enable automated risk profiling [21,22]. Randomized trials could also test whether reductions in DDRS translate into measurable improvements in caries or periodontal disease. If validated, DDRS could become an important bridge between nutritional epidemiology and dental practice, advancing personalized and preventive oral healthcare.

## Conclusions

The DDRS represents a novel conceptual framework for integrating dietary patterns with oral health risk. By explicitly modeling sugar, acidic beverages, protective foods, and overall quality, DDRS bridges nutrition indices with dental outcomes. Its strong inverse correlations with MEDAS, DPI, and HEI highlight its potential. Sugar remains the dominant determinant of dental risk, but protective foods and general dietary quality also play important roles. However, as this model is based on a synthetic dataset and theoretical weight assignments, its findings should be interpreted conceptually rather than empirically. DDRS warrants validation in clinical cohorts and could become a potentially valuable framework for preventive dentistry. In addition to advancing theoretical modeling, this framework may guide clinicians in identifying patients at higher dietary risk for oral diseases. The DDRS could also serve as a bridge between nutrition science and dentistry, fostering more integrated preventive strategies. Overall, this study lays the groundwork for future validation efforts in diverse populations.
